# Human umbilical cord-derived mesenchymal stem cells alleviate insulin resistance in diet-induced obese mice via an interaction with splenocytes

**DOI:** 10.1186/s13287-022-02791-6

**Published:** 2022-03-21

**Authors:** Jing Xue, Jieqing Gao, Yulin Gu, Aihong Wang, Songyan Yu, Bing Li, Yaqi Yin, Jie Wang, Wanlu Su, Haixia Zhang, Weizheng Ren, Weijun Gu, Zhaohui Lv, Yiming Mu, Yu Cheng

**Affiliations:** 1 Medical School of Chinese PLA, Beijing, China; 2 Department of Endocrinology, The First Medical Center of Chinese PLA General Hospital, Beijing, China; 3 Department of Endocrinology, Diabetes Center of People’s Liberation Army (PLA), PLA Strategic Support Force Characteristic Medical Center (The 306th Hospital of PLA), Beijing, China; 4 Department of Endocrinology, Beijing Rehabilitation Hospital, Capital Medical University, Beijing, China; 5 Department of Endocrinology, Beijing Tiantan Hospital, Capital Medical University, Beijing, China; 6 School of Medicine, Nankai University, Tianjin, China

**Keywords:** Insulin resistance, Obesity, Spleen, Mesenchymal stem cells, Macrophage, Regulatory T cell

## Abstract

**Background:**

Previous research has demonstrated that the spleen plays an important role in mesenchymal stem cell (MSC)-mediated alleviation of acute inflammation, as MSC infusion increases the spleen-derived anti-inflammatory cytokine interleukin 10 (IL-10) levels. However, studies on splenic involvement in MSC-induced protection against chronic inflammatory diseases are limited. Obesity is characterized by chronic low-grade inflammation, a key driver of insulin resistance. This study aims to evaluate the effects of MSCs on obesity-related insulin resistance and explore the underlying mechanism, particularly regarding splenic involvement.

**Methods:**

We induced obesity in mice by feeding them high-fat diets for 20 weeks. Human umbilical cord-derived MSCs (UC-MSCs) were systemically infused into the obese mice once per week for 6 weeks. Systemic glucose metabolic homeostasis and insulin sensitivity in epididymal adipose tissue (EAT) were evaluated. Then, we conducted in vivo blockade of IL-10 during UC-MSC infusion by intraperitoneally administrating an IL-10-neutralizing antibody twice per week. We also investigated the therapeutic effects of UC-MSCs on obese mice after removal of the spleen by splenectomy.

**Results:**

UC-MSC infusions improved systemic metabolic homeostasis and alleviated insulin resistance in EAT but elicited no change in weight. Despite rare engraftment of UC-MSCs in EAT, UC-MSC infusions attenuated insulin resistance in EAT by polarizing macrophages into the M2 phenotype, coupled with elevated serum IL-10 levels. In vivo blockade of IL-10 blunted the effects of UC-MSCs on obese mice. Furthermore, UC-MSCs overwhelmingly homed to the spleen, and the ability of UC-MSCs to elevate serum IL-10 levels and alleviate insulin resistance was impaired in the absence of the spleen. Further in vivo and in vitro studies revealed that UC-MSCs promoted the capacity of regulatory T cells (Treg cells) to produce IL-10 in the spleen.

**Conclusions:**

Our results demonstrated that UC-MSCs elevated serum IL-10 levels and subsequently promoted macrophage polarization, leading to alleviation of insulin resistance in EAT. The underlying mechanism was that UC-MSCs improved the capacity of Treg cells to produce IL-10 in the spleen. Our findings indicated that the spleen played a critical role in amplifying MSC-mediated immunomodulatory effects, which may contribute to maximizing MSC efficacy in clinical applications in the future.

**Supplementary Information:**

The online version contains supplementary material available at 10.1186/s13287-022-02791-6.

## Introduction

Obesity, characterized by chronic low-grade inflammation in white adipose tissue (WAT), plays a central role in the onset and progression of insulin resistance, which contributes to a series of life-threatening diseases, such as type 2 diabetes mellitus (T2DM) and cardiovascular disease [1, 2].

Research has identified macrophages as the main effectors in obesity-induced adipose inflammation [1, 3]. Macrophages are typically classified into classically activated pro-inflammatory M1 macrophages and alternatively activated anti-inflammatory M2 macrophages, although the dichotomy is an oversimplification [4, 5]. In lean individuals, most resident macrophages in WAT are M2 macrophages, marked by the expression of CD206, found in inflammatory zone 1 (Fizz1), and arginase-1 (Arg1) [6]. However, in obese individuals, the accumulation of macrophages in WAT increases [7] along with macrophage polarization towards the M1 phenotype, with increased expression of CD11c, inducible nitric oxide synthase (iNOS) and tumour necrosis factor (TNF)-α, promoting the inflammatory process and leading to insulin resistance in WAT [6, 8, 9]. Remodelling the balance of M1/M2 macrophages in WAT is recognized to be a new therapeutic target to alleviate insulin resistance.

Mesenchymal stem cells (MSCs) are pluripotent self-renewable stem cells. Compelling evidence has shown that in various inflammatory disease models, MSC infusion facilitates macrophage polarization into M2 macrophages, thereby suppressing inflammation and improving the function of targeted organs [10–12]. Our previous research demonstrated that human umbilical cord-derived MSC (UC-MSC) alleviated insulin resistance in T2DM mice by stimulating macrophages polarization towards the M2 phenotype in WAT. Some studies have documented that MSCs preferentially engraft inflammatory tissues and exert anti-inflammatory effects through paracrine mechanisms [13]. However, our previous research demonstrated that MSCs rarely home to WAT [14, 15]. Therefore, how can MSCs modulate the phenotype of macrophages with scarce engraftment in WAT?

Interestingly, it is reported that MSCs could exert protective effects in mouse models of acute kidney injury [16], traumatic spinal cord injury [17] and stroke [18, 19] despite rare homing to the injured organ, and the underlying mechanism is modulating immune cells in the spleen. For instance, Anna Badner and colleagues found that UC-MSC infusion alleviated spinal cord injury and increased systemic levels of interleukin-10 (IL-10), but these UC-MSC-induced changes were abolished by splenectomy, indicating that the spleen was a crucial target of MSC therapy. [17]. Intriguingly, our previous study also revealed that in a T2DM mouse model, MSCs overwhelmingly homed to the spleen after intravenous infusion [20]. Therefore, we speculate that MSCs promote macrophage polarization in WAT via interaction with splenocytes.

IL-10, a potent anti-inflammatory cytokine, is capable of polarizing macrophages towards the M2 phenotype [21, 22]. Growing evidence has shown that low serum levels of IL-10 are closely correlated with insulin resistance [23, 24], while elevating serum IL-10 levels via exogenous IL-10 administration promotes amelioration of insulin resistance by skewing macrophages to M2 in high-fat diets (HFD)-fed mice [25, 26]. Notably, studies have revealed that the reduction in serum IL-10 levels in obese individuals probably results from the decreased production of IL-10 from the spleen, implying that spleen-derived IL-10 plays a vital role in the prevention of obesity-induced chronic inflammation [27, 28]. Of note, our previous study on T2DM mice found that after UC-MSC infusion, IL-10 expression in the spleen remarkably increased. Accordingly, we postulated that UC-MSCs could polarize macrophages towards M2 to alleviate insulin resistance in WAT by upregulating IL-10 production in the spleen.

In this study, we investigated the effect and underlying mechanisms of UC-MSC on insulin resistance in HFD-induced obese mice. We reported that multiple UC-MSC infusions polarized macrophages in WAT into the M2 phenotype and attenuated insulin resistance in HFD mice. The underlying mechanism involved the UC-MSC-induced increase in serum IL-10 levels, which were attenuated by splenectomy. Further investigation unveiled that MSCs promoted IL-10 production by splenic regulatory T cells (Treg cells) cells both in vivo and in vitro, highlighting that the spleen was a crucial target of systemically infused UC-MSCs.

## Methods

### Animal experiment

Eight-week-old male C57BL/6J mice were obtained from Chinese PLA General Hospital. Five mice were housed per cage under standard conditions (free access to food and water, 12 h/12 h light-and-dark cycle and constant room temperature of 25 °C). Mice in the same cage were distinguished by the metal label tagged on ear. Mice were fed HFD for 20 weeks to induce obesity, and mice fed a normal chow diet were used as controls (referred to as the Nor group). The HFD contained 5.24 kcal/g and was composed of 60% kcal% fat, 20% kcal% protein and 20% kcal% carbohydrate (D12492, Research Diets, New Brunswick, NJ). The normal diet contained 3.40 kcal/g and was composed of 11.85% kcal% fat, 23.07% kcal% protein and 65.08% kcal% carbohydrate (BEIJING KEAO XIELI FEED CO, Beijing, China). Obese mice were randomly divided into the HFD group or the MSC group. The mice of MSC group were injected with 1 × 10^6^ UC-MSCs (passage 4)suspended in 0.2 mL of phosphate-buffered saline (PBS) via the tail vein once per week for 6 consecutive weeks, and the mice of HFD group were injected with PBS alone. After the last infusion of UC-MSCs, intraperitoneal glucose tolerance tests (IPGTTs) and insulin tolerance tests (IPITTs) were performed. For IPITTs, mice were fasted for 6 h and intraperitoneally injected with insulin (0.75 U/kg). For IPGTTs, mice were fasted overnight and intraperitoneally injected with glucose (1.75 g/kg). Blood glucose levels were detected via tail vein blood at 0, 30, 60, 90 and 120 min by a glucometer (Sinocare, Changsha, China) after the injection. Blood glucose levels were measured. Fasting blood insulin was quantified by an enzyme-linked immunosorbent assay (ELISA) kit (Mercodia, Uppsala, Sweden), and the homeostatic model assessment for insulin resistance (HOMA-IR) was calculated based on the following equation: (fasting blood glucose level [in mmol/L] × fasting serum insulin level [in mIU/L])/22.5.

To explore the homing of UC-MSCs, obese mice were infused with chloromethyl-benzamidodialkylcarbocyanine (CM-Dil) (C7000, Life Technologies, Eugene, Oregon, USA)-labelled UC-MSCs (passage 4) via the tail vein, and then they were sacrificed at 6 h, 12 h, 24 h, 3 d and 7 d after infusion. The procedure of sacrificing mice was to anesthetize mice with 1% pentobarbital sodium (50 mg/kg) and then perfuse 20 ml PBS through the left ventricle followed with 15 ml 4% paraformaldehyde.

### In vivo neutralization of IL-10

C57BL/6 mice were fed a HFD for 20 weeks to induce obesity and then infused with 1 × 10^6^ UC-MSCs (passage 4) once per week for 6 consecutive weeks. During UC-MSC treatment, mice were intraperitoneally injected with a neutralizing anti-IL-10 antibody (200 μg/mouse) or the corresponding isotype control IgG twice per week.

### Splenectomy

Obese mice were anaesthetized with intraperitoneal injection of sodium pentobarbital (50 mg/kg). A small incision was made in the left upper quadrant of the abdomen. Then, the spleen was carefully removed after cutting the connective ligament and ligating splenic vessels. The incision was closed carefully with a suture. For sham-operated mice, incisions were made and sutured without removal of the spleen. All the mice received intraperitoneal antibiotic administration during the operation and rested for 3 weeks before further experimental procedures. Sham-operated were infused with 1 × 10^6^ UC-MSCs (passage 4) (referred to as the SHAM + MSC group) or PBS (referred as the SHAM group) once per week for 6 consecutive weeks. In parallel, mice that underwent splenectomy were infused with 1 × 10^6^ UC-MSCs (passage 4)(referred to as the SPX + MSC group) or PBS (referred as the SPX group) once per week for 6 consecutive weeks.

### Cell culture

UC-MSCs were isolated from human umbilical cords that were obtained from women who gave birth at Chinese PLA General Hospital. UC-MSCs were isolated, purified and identified as described previously [29, 30]. Briefly, human umbilical cords were freshly obtained with donor consent and then were washed with sterile PBS to remove the cord blood. Next, the umbilical artery, umbilical vein, and the connective tissue of umbilical cords were removed and the remaining cord tissue was cut into 1 mm^3^ pieces. Then, the pieces were directly placed into culture dishes and the growing medium of Dulbecco's modified Eagle's medium with 10% UltraGRO™-Advanced (HPCFDCRL50, HELIOS Bioscience, USA) were added into the dishes. When cells reached subconfluence, tissue pieces were removed and cells adhering to the dishes were digested and passaged. Passage 4 of UC-MSCs was identified and used in the following experiment. UC-MSCs were cultured in an incubator set at 37 °C in a 5% CO_2_ environment.

The spleens were removed aseptically from euthanized obese mice. The spleens were washed once with PBS, minced into small pieces in RPMI 1640 medium and then ground gently to release splenocytes. The effluent samples were filtered through a 200 μm filter and centrifuged at 1000 × g for 5 min. The supernatant was discarded, and the pellet was suspended in 3 mL of red blood cell lysis buffer and incubated for 4 min according to the manufacturer’s protocols. Subsequently, the samples were washed twice with RPMI 1640 medium. Before seeding onto six-well plates, splenocytes were counted and assessed for cell viability through Trypan blue exclusion. Splenocytes were cultured in RPMI 1640 medium supplemented with 10% foetal calf serum (Gibco, CA, USA), 1% penicillin streptomycin (Gibco, CA, USA), 50 mmol/L 2-mercaptoethanol (Gibco, CA, USA), 10 mmol/L *N*-2-hydroxyethylpiperazine-*N*’-2-ethanesulfonic acid (HEPES, Gibco, CA, USA), and 10 U/ml low-dose recombinant mouse interleukin-2 (PeproTech China, Suzhou, China) at 37 °C in 5% CO_2_.

Splenocytes (1 × 10^6^ cells) were cultured alone or in co-culture with UC-MSCs (4 × 10^4^ cells) in a Transwell system for 24 h. Then, UC-MSCs were removed, and splenocytes were activated with concanavalin A (Con A) for 72 h. Next, the expression of CD4, CD25, Foxp3 and IL-10 in splenocytes was detected by flow cytometry, and the protein levels of IL-10 in the supernatant were determined by ELISA.

### Western blotting

Mouse epididymal adipose tissue (EAT) was lysed in Tissue Protein Extraction Reagent (CWBIO, China) with 1% protease inhibitor cocktail (CWBIO, China) and 10% phosphatase inhibitors (Roche, Switzerland). The protein concentration was determined by BCA assay (CWBIO, China). The proteins were loaded onto SDS-PAGE gels (GeneScript, China) and then transferred to PVDF membranes (EMD Millipore). Subsequently, the membranes were blocked with 10% bovine serum albumin (BSA) or 5% milk and then incubated with primary antibodies against Arg-1 (1:1000, Abcam, USA), p-AKT (1:1000, CST, USA), AKT (1:1000, CST, USA), β-actin (1:2000, ZSJQ-BIO, China), and β-actin (1:2000, ZSJQ-BIO, China) overnight at 4 °C, followed by incubation with secondary antibodies for 1 h at room temperature. The secondary antibodies included goat anti-rabbit and goat anti-mouse IgG HRP-conjugated antibodies (ZSJQ-BIO, China). The blots were visualized with an ECL detection system (PPLYGEN, China) and analysed using ImageJ software (NIH, USA).

### Flow cytometry analysis

Splenocytes harvested from mice of the Nor group, HFD group and MSC group were incubated with APC-conjugated anti-Foxp3 antibody (eBioscience, USA), FITC-conjugated anti-CD4 antibody, BV421-conjugated anti-CD3 antibody, APC/Cyanine7-conjugated anti-CD8 antibody, BV510-conjugated anti-NK1.1 antibody, APC/Cyanine7-conjugated anti-Ly6G antibody, APC-conjugated anti-Ly6C antibody, BV510-conjugated anti-CD11b antibody, FITC-conjugated anti-F4/80 antibody, PE/Cy7-conjugated anti-CD11c antibody, BV421-conjugated anti-CD206 antibody, APC/Cyanine7-conjugated anti-B220 antibody (Biolegend, USA), BV421-conjugated anti-CD25 antibody, PE-Cy7-conjugated anti-CD19 antibody, FITC-conjugated anti-CD5 antibody, Alexa Fluor® 647-conjugated anti-CD1d antibody, and PE-conjugated anti-IL-10 antibody (BD, CA). Corresponding isotype antibodies were used as negative controls. An intracellular fixation and a permeabilization buffer set (eBioscience) were used for intracellular staining.

In the in vitro study, freshly harvested splenocytes were washed twice and then incubated with FITC-conjugated anti-CD4 antibody, BV421-conjugated anti-CD25 antibody, APC-conjugated anti-Foxp3 antibody and PE-conjugated anti-IL-10 antibody. Raw data were analysed using Kaluza analysis software (Beckman Coulter, USA).

### RNA extraction and real-time PCR (RT-PCR) analysis

Total RNA was extracted from mouse EAT and splenocytes using TRIzol Reagent (Invitrogen), and its concentration was determined by a NanoDrop system (Thermo Fisher Scientific, Waltham, MA) according to the manufacturer’s instructions. Next, RNA was converted into cDNA with a Fast All-in-One RT Kit (ES Science, Shanghai, China), and real-time polymerase chain reaction (RT-PCR) was performed using a 7500 Real-Time PCR System with Super SYBR Green PCR Master Mix (ES Science, Shanghai, China). The thermal cycling protocol was 94 °C for 3 min, followed by 94 °C for 30 s, 60 °C for 30 s and 72 °C for 30 s for 40 cycles. Gapdh was used as an internal reference. The sequences of the RT-PCR primers are listed in Additional file 1: Table S1.

### Histopathology and immunofluorescence staining

Mice were anaesthetized with intraperitoneal injection of sodium pentobarbital and then underwent consecutive perfusions of 20 ml of PBS and 20 ml of 4% paraformaldehyde through the right ventricle. For H&E staining, EAT was embedded in paraffin, sectioned at 10 μm thickness and then stained with H&E according to the standard protocol. For immunofluorescence staining, EAT was dehydrated through incubation in 30% sucrose/PB overnight, embedded in optimal cutting temperature compound (OCT, Sakura, Finetek, USA) and cut into frozen sections at 10 μm. The frozen sections were blocked with BSA and then incubated at 4 °C overnight with indicated primary antibodies against Fizz1 (ab39626, Abcam, San Francisco, USA), iNOS (ab15323, Abcam, San Francisco, USA) and F4/80 (sc-377009, Santa Cruz, USA), followed by further incubation with Alexa Fluor 488/594-conjugated secondary antibodies (1: 500, Life Technologies), shielding from light, at room temperature for 2 h. Finally, the sections were incubated with 4′-6-diamidino-2-phenylindole (DAPI) for 7 min for nuclear staining and imaged with a laser scanning confocal microscope (Leica, Wetzlar, Germany).

### Cytokine assays

Mouse serum levels of interleukin 1β (IL-1β), TNF-α, interleukin 6 (IL-6), IL-10, and monocyte chemoattractant protein-1 (MCP-1) were determined by AimPlex™ assay kits (QuantoBio, Beijing, China) according to the manufacturer’s instructions. Removed spleens and EAT were homogenized, and supernatants were collected after centrifugation. Then, the concentrations of IL-10 in the supernatants were detected by AimPlex™ assay kits. The secretion of IL-10 from splenocytes was quantified using ELISA.

### Statistics

Data were analysed using GraphPad Prism 6 software. All values are expressed as the means ± SDs from at least three independent samples. Differences between two groups were assessed using 2-tailed Student’s t test or the Mann–Whitney U test, while differences between multiple groups were evaluated by 1-way ANOVA or the Kruskal–Wallis test as appropriate. A two-tailed *P* < 0.05 was considered statistically significant.

### Study approval

All experimental protocols were approved by the Ethics Committee of Chinese PLA General Hospital, and samples from human tissues were obtained with written informed consent from the donors. All animals were handled in accordance with the *Guide for the Care and Use of Laboratory Animals* of the National Institutes of Health (National Academies Press, 2011) in China.

## Results

### Multiple UC-MSC infusions resulted in a subtle improvement in systemic metabolic homeostasis in HFD-fed mice

**Fig. 1:**
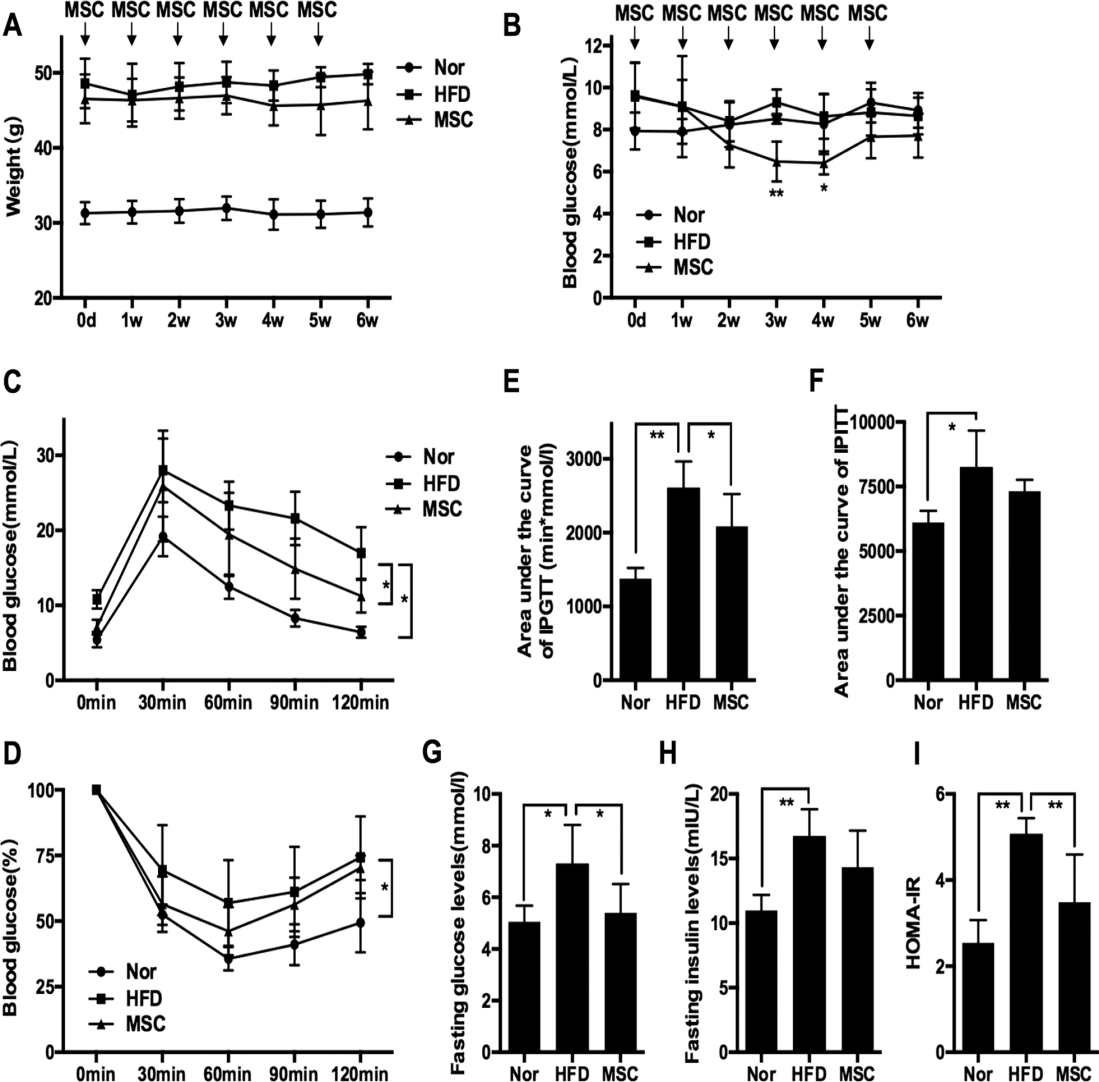
Multiple UC-MSC infusions resulted in a subtle improvement in systemic metabolic homeostasis in HFD-fed mice. Eight-week-old male C57BL/6J mice were fed a HFD for 20 weeks to induce obesity. Then, the obese mice were randomly treated with an infusion of 0.2 ml of PBS (referred to as the HFD group) or an infusion of 1 × 10^6^ UC-MSCs suspended in 0.2 ml of PBS once per week for 6 weeks (referred to as the MSC group). Mice fed a normal chow diet were used as controls (referred to as the Nor group). One week after the last infusion of UC-MSCs, the mice were sacrificed. The weight (**a**) and random blood glucose levels (**b**) of mice were measured once per week. After the last infusion of UC-MSCs, glucose tolerance and insulin tolerance were assessed by an IPGTT (**c**) and IPITT (**d**), respectively. **e** The area under the curve of the IPGTT was calculated. **f** The area under the curve of the IPITT was calculated. The fasting glucose levels (**g**) and the fasting insulin levels (**h**) were detected after the last infusion of UC-MSCs. The HOMA-IR score was calculated (**i**). Values of **a**–**i** are the means ± SDs; *n* = 6 mice per group; *P < 0.05, **P < 0.01. UC-MSCs, human umbilical cord-derived mesenchymal stem cells; HFD, high-fat diets; PBS phosphate-buffered saline; IPGTT, intraperitoneal glucose tolerance test; IPITT, intraperitoneal insulin tolerance test

First, we evaluated the effect of UC-MSCs on obesity and systemic metabolic homeostasis in HFD-fed mice. Compared with the chow diet, the HFD induced marked weight gain in mice (Additional file 1: Fig. 1A). Long-term HFD resulted in insulin resistance and glucose intolerance (Additional file 1: Fig. 1 B, C). Multiple UC-MSC infusions resulted in a decreasing trend in body weight, but the difference did not reach statistical significance (Fig. 1A). Random blood glucose levels of the MSC group were significantly reduced after the third and fourth UC-MSC infusions but fluctuated to a level similar to that of the HFD group after the fifth UC-MSC infusion (Fig. 1B). Of note, significant improvement in glucose tolerance was found in HFD-fed mice treated with UC-MSCs, as demonstrated by IPGTT results and IPGTT areas of under the curve (Fig. 1C, E). The IPITT results showed an increasing tendency of insulin sensitivity after UC-MSC infusions (Fig. 1D). The fasting blood glucose levels of the MSC group decreased compared with that of the HFD group (Fig. 1G). The mice in the MSC group also showed a decreasing trend in the IPITT areas of under the curve (Fig. 1F), fasting insulin level (Fig. 1H) and HOMA-IR score (Fig. 1I), although there were no statistically significant differences between the HFD group and MSC group. These results indicated that UC-MSC infusion led to subtle amelioration of systemic metabolic homeostasis in HFD-fed mice.

### Multiple UC-MSC infusions alleviated insulin resistance and inflammation of EAT in HFD-fed mice

**Fig. 2:**
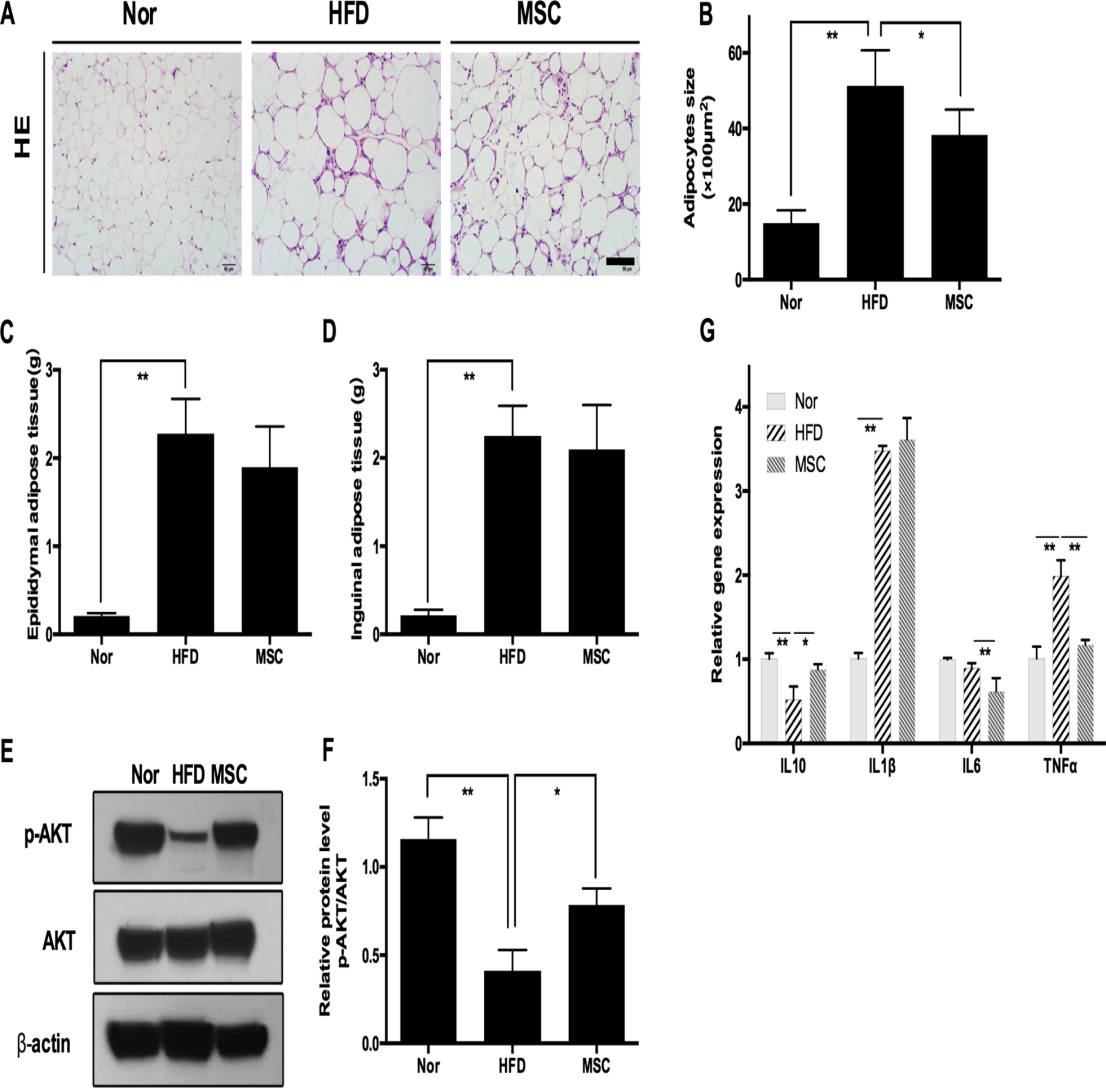
Multiple UC-MSC infusions alleviated insulin resistance and inflammation of epididymal adipose tissue in HFD-fed mice. **a** Representative H&E staining of epididymal adipose tissue from the Nor, HFD and MSC groups. Scale bar = 100 μm. **b** The size of adipocytes was evaluated via digital image analysis. **c** Weight of inguinal adipose tissue. **d** Weight of epididymal adipose tissue. **e** Immunoblotting analysis of p-AKT and total AKT expression in epididymal adipose tissue. The ratios of p-AKT to total AKT were quantitated (**f**). **g** Real-time polymerase chain reaction analysis of inflammation-related gene expression in epididymal adipose tissue. The results are presented relative to those of normal mice, which were set as 1. Values are the mean ± SD; *n* = 6 mice per group, *P < 0.05; **P < 0.01

Adipose tissue plays a vital role in the maintenance of metabolic homeostasis. We next assessed the morphological and functional alteration of EAT, a critical component of visceral adipose tissue. Despite the subtle effect of UC-MSCs on systemic metabolic homeostasis, H&E staining (Fig. 2A) revealed that UC-MSC infusion dramatically attenuated HFD-induced adipocyte hypertrophy of EAT, as demonstrated by the decreasing average size of adipocytes after UC-MSC infusions (average adipocyte sizes of the HFD group and MSC group, 5116 μm^2^ vs. 3828 μm^2^, *P* < 0.05) (Fig. 2B). Simultaneously, compared with mice in the HFD group, mice in the MSC group showed a drastic elevation in p-AKT expression (Fig. 2E, F), indicating an obvious alleviation of insulin resistance in EAT, although UC-MSC treatment did not exert an impact on the weight of either subcutaneous tissue or EAT (Fig. 2C, D). Given that inflammation plays an essential role in the onset and progression of insulin resistance, we examined the inflammatory status of EAT. RT-PCR displayed increased expression of the gene encoding IL-10, a critical anti-inflammatory molecule, and decreased expression of genes encoding pro-inflammatory molecules, including IL-6 and TNF-α (Fig. 2G). Collectively, these results implied that multiple UC-MSC infusions were effective in attenuating insulin resistance and inflammation in EAT.

### Multiple UC-MSC infusions promoted macrophage polarization towards the M2 phenotype in EAT

**Fig. 3:**
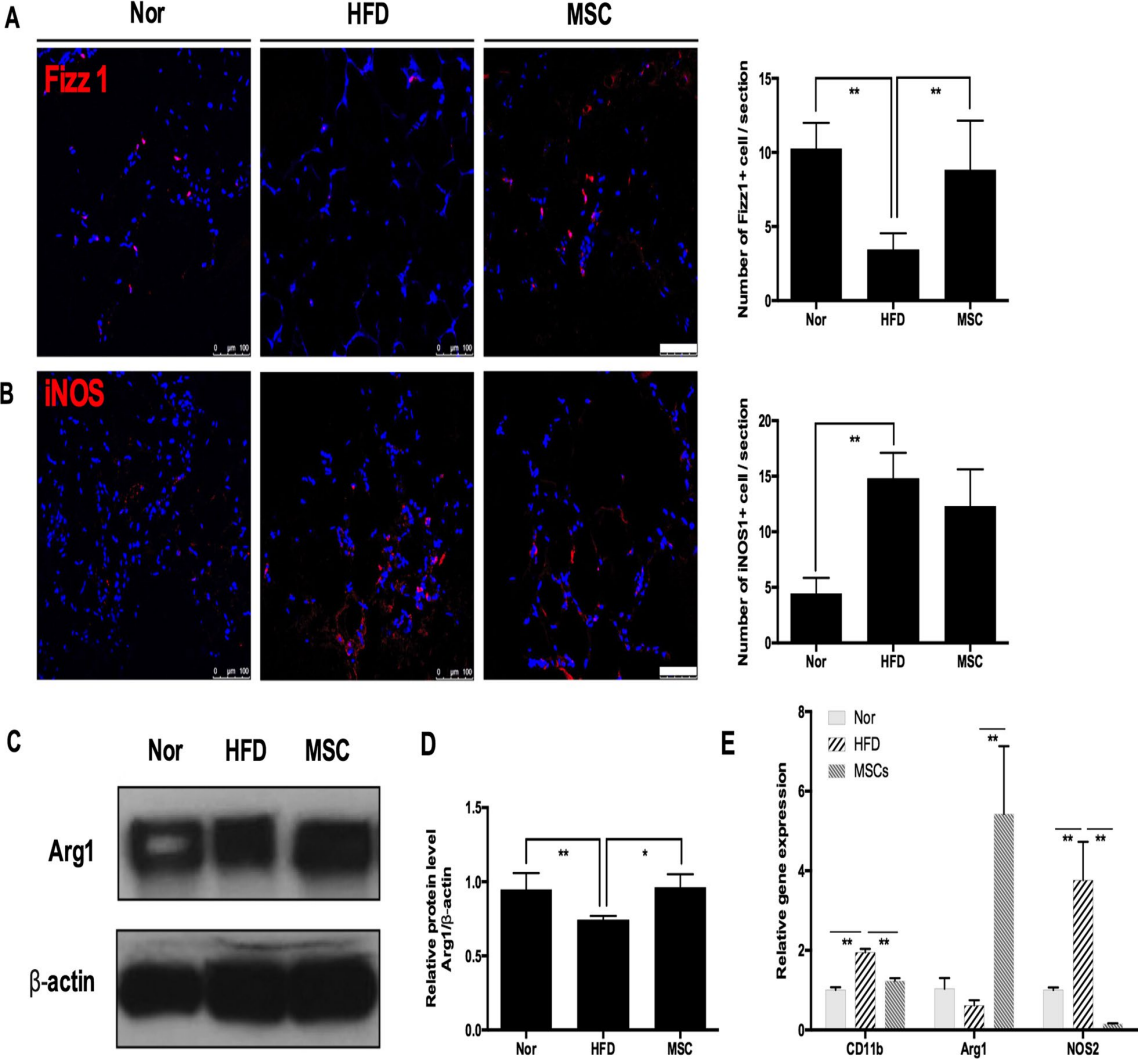
Multiple UC-MSC infusions promoted macrophage polarization towards the M2 phenotype in epididymal adipose tissues. **a** Representative Fizz1-positive cells (M2 macrophage marker) in epididymal adipose tissue analysed by immunofluorescence and quantification of Fizz1-positive cells. Scale bar = 100 μm. **b** Representative iNOS-positive cells (M1 macrophage marker) in epididymal adipose tissue analysed by immunofluorescence and quantification of iNOS-positive cells. Scale bar = 100 μm. Values in **a** and **b** were determined by assessing cells manually from at least five sections of each slide, at least 3 slides per mouse, and at least 6 mice per group. **c** Immunoblotting analysis of Arg1 expression in epididymal adipose tissue. Relative protein levels were quantified by the ratio of Arg1 to β-actin (**d**). **e** Real-time polymerase chain reaction analysis of macrophage phenotype-related gene expression in epididymal adipose tissue. The results are presented relative to those of normal mice, which were set as 1. Values are the mean ± SD; *n* = 6 mice per group, *P < 0.05; **P < 0.01

Previous research has confirmed that M2 macrophages are an important contributor to the maintenance of insulin sensitivity; therefore, we next explored the effect of UC-MSCs on macrophages in EAT. Similar to previous research, we noted that the HFD elicited macrophage polarization towards M1 in EAT, as evidenced by more iNOS-positive cells (M1 macrophage marker) (Fig. 3B) and fewer Fizz1-positive cells (M2 macrophage marker) (Fig. 3A). Interestingly, the number of Fizz1-positive cells in the MSC group increased dramatically in comparison with the HFD group (*P* < 0.01), while the number of iNOS-positive cells in the MSC group show a descending trend in comparison with the HFD group (*P* = 0.23). Western blotting also showed that UC-MSC infusions evoked elevated expression of Arg-1, a typical marker of M2 macrophages. Furthermore, UC-MSC infusion upregulated the mRNA level of Arg1 (Fig. 3C, D) while decreasing the mRNA levels of iNOS and CD11b (Fig. 3E). Taken together, these data suggested that multiple UC-MSC infusions induced macrophage polarization towards the M2 phenotype in EAT.

### IL-10 neutralization attenuated UC-MSC-induced alleviation of insulin resistance in EAT

To further delineate the underlying mechanism of UC-MSC-induced alleviation of insulin resistance in EAT, we explored the homing of UC-MSCs by staining UC-MSCs with CM-Dil. Intriguingly, we found very few CM-Dil-positive cells in EAT. The engraftments of MSCs in pancreas, thymus and bone marrow were also limited. A small proportion of CM-Dil-positive cells existed in the lung and liver soon after infusion, but it decreased to a low level 3 days later. Notably, a large number of CM-Dil-positive cells continuously existed in the spleen throughout the experimental course (Additional file 1: Fig. 2A-E). Due to the rare homing of UC-MSCs to EAT, we speculated that UC-MSCs polarized macrophages by an indirect mechanism rather than a direct effect on macrophages.

**Fig. 4:**
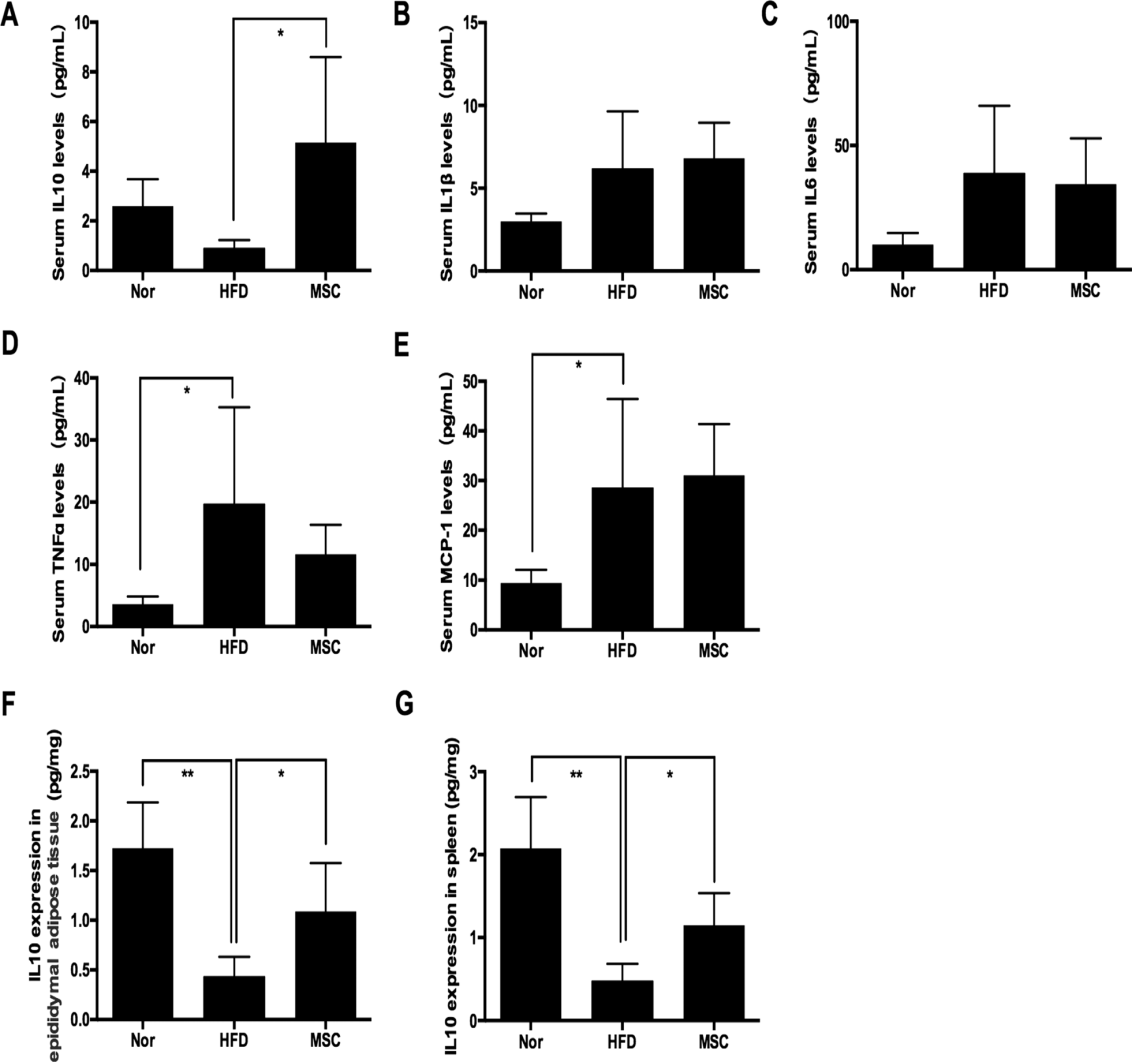
Multiple UC-MSC infusions elevated the levels of IL-10 in serum, adipose tissue and the spleen. The serum levels of IL-10 (**a**), IL-1β (**b**), IL-6 (**c**), TNF-α (**d**) and MCP-1 (**e**) were determined by AIMPLEX assay. IL-10 protein expression in epididymal adipose tissue (**f**) and the spleen (**g**) was measured by ELISA. Values are the mean ± SD; *n* = 6 mice per group, *P < 0.05; **P < 0.01

IL-10 has been identified as a potent anti-inflammatory cytokine that can polarize macrophages into M2 macrophages. Interestingly, we found that the serum concentration of IL-10, which was suppressed by the HFD, was elevated by UC-MSC infusion, while there was no significant difference in serum levels of IL-1β, IL-6, TNF-α or MCP-1 between the HFD group and MSC group (Fig. 4A–E). Our abovementioned results showed an increase in the levels of IL-10 mRNA in EAT after UC-MSC infusions (Fig. 2G), and further exploration by ELISA showed that the protein level of IL-10 in EAT also increased (Fig. 4F). Furthermore, UC-MSC infusion upregulated the protein level of IL-10 in the spleen (Fig. 4G), where the largest amount of UC-MSCs homed.

Based on the results, we speculated that IL-10 probably contributed to the effect of UC-MSCs on insulin resistance in EAT. To substantiate this hypothesis, we infused UC-MSCs into mice that were fed a HFD for 20 weeks coupled with intraperitoneal administration of an IL-10-neutralizing antibody (MSC + IL-10Ab group) to determine whether the therapeutic effect of UC-MSCs was impaired.

**Fig. 5:**
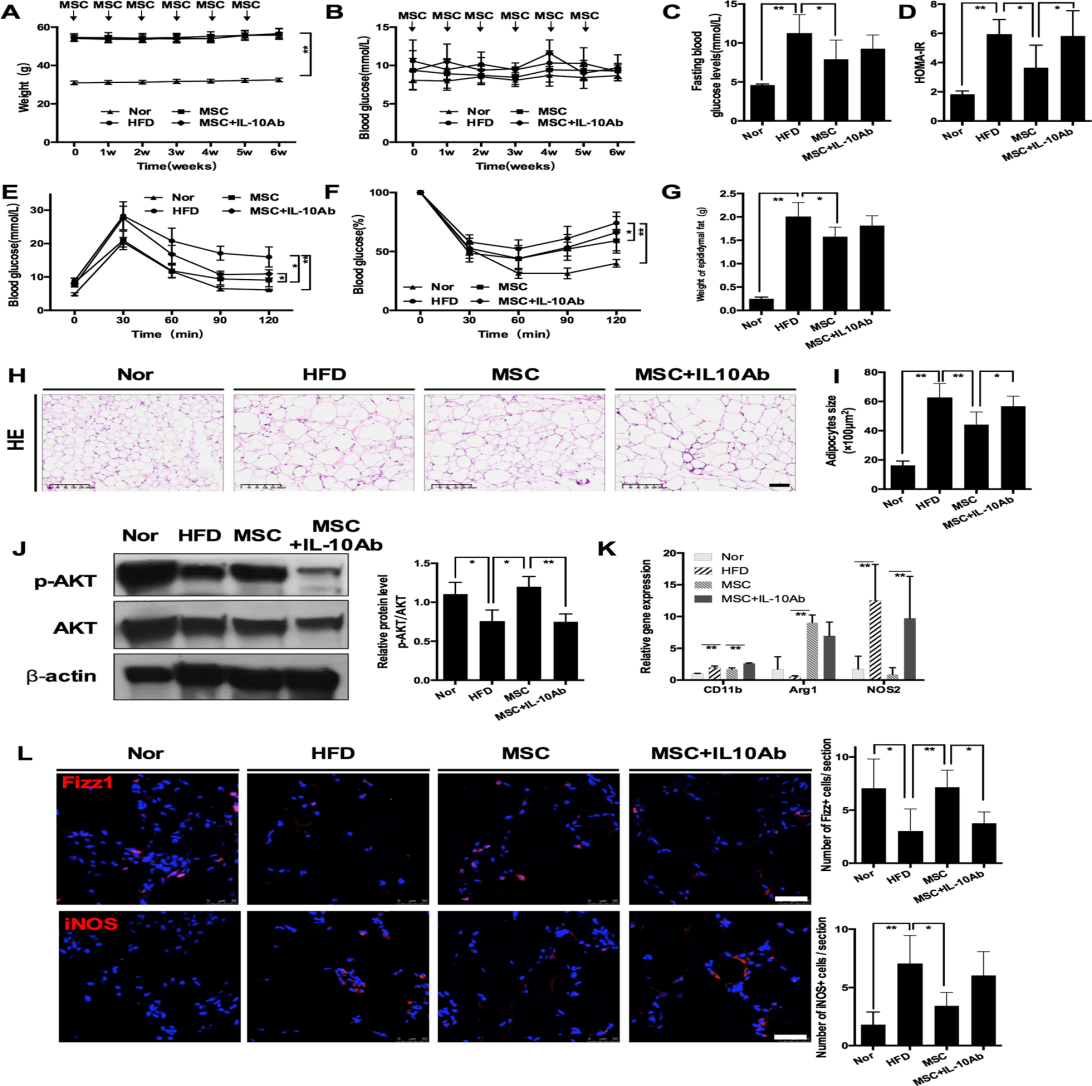
IL-10 neutralization attenuated UC-MSC-induced alleviation of insulin resistance and macrophage polarization in epididymal adipose tissue. HFD-induced obese mice were randomly divided into the HFD group, MSC group and MSC + IL-10Ab group. The MSC group and MSC + IL-10Ab group both received UC-MSC infusions once per week for 6 weeks. During UC-MSC infusion, the MSC + IL-10Ab group and MSC group were intraperitoneally administered a neutralizing anti-IL-10 antibody or the corresponding isotype control IgG twice per week. The HFD group received PBS infusions. Mice fed a normal chow diet were used as controls (referred to as the Nor group). One week after the last infusion of UC-MSCs, the mice were sacrificed. **a** Weight of the mice. **b** Random blood glucose levels of the mice. **c** The fasting glucose levels after the last infusion of UC-MSCs were detected. The HOMA-IR score was calculated (**d**). After the last infusion of UC-MSCs, glucose tolerance and insulin tolerance were assessed by an IPGTT (**e**) and IPITT (**f**), respectively. **g** Weight of epididymal adipose tissue. **h** Representative H&E staining of epididymal adipose tissue from the Nor, HFD, MSC and MSC + IL-10Ab groups. Scale bar = 100 μm. **i** The size of adipocytes was evaluated via digital image analysis. **j** Immunoblotting analysis of p-AKT and total AKT expression in epididymal adipose tissue; the ratios of p-AKT to total AKT were quantitated. **k** Real-time polymerase chain reaction analysis of macrophage phenotype-related gene expression in epididymal adipose tissue. **l** Representative Fizz1-positive cells and iNOS-positive cells in epididymal adipose tissue analysed by immunofluorescence and quantification of Fizz1-positive or iNOS-positive cells. Scale bar = 50 μm. Values in **l** were determined by assessing cells manually from at least five sections of each slide, at least 3 slides per mouse, and at least 6 mice per group. Values of **a**–**h** are the means ± SDs; *n* = 6 mice per group; *P < 0.05, **P < 0.01

Similar to previous results, prolonged MSC infusion resulted in subtle alleviation of systemic metabolic homeostasis (Fig. 5A–F). The anti-IL-10 antibody dampened the beneficial effect of MSCs on systemic metabolic homeostasis, as demonstrated by higher blood glucose levels during the IPGTT and an ascending trend in HOMA-IR scores in the MSC + IL-10Ab group in comparison with those in the MSC group (Fig. 5D, E). The analyses of EAT showed that compared with the MSC group, the MSC + IL-10Ab group presented comparable weights of EAT (Fig. 5G) but enlarged adipocytes (Fig. 5H, I), lower expression of p-AKT (Fig. 5J). Moreover, compared with EAT of the MSC group, there were more M1 macrophages (*P* = 0.08) and fewer M2 macrophages (*P* < 0.05) in the EAT of the MSC + IL-10Ab group (Fig. 5K, L). In summary, the anti-IL-10 antibody blunted UC-MSC-induced macrophage polarization and amelioration of insulin resistance in EAT. These results revealed that the increasing level of IL-10 elicited by UC-MSC infusion played an important role in the therapeutic effects of UC-MSCs.

### Splenectomy dampened UC-MSC-induced mitigation of insulin resistance in EAT

Given the overwhelming homing of UC-MSCs in HFD-fed obese mice along with an increase in IL-10 levels in the spleen, we hypothesized that the spleen was critical in the UC-MSC-induced increase in systemic IL-10 levels. To test this hypothesis, we performed a splenectomy or sham operation on HFD-fed mice. After resting for a sufficient period to recover, the mice were administered UC-MSCs.

**Fig. 6:**
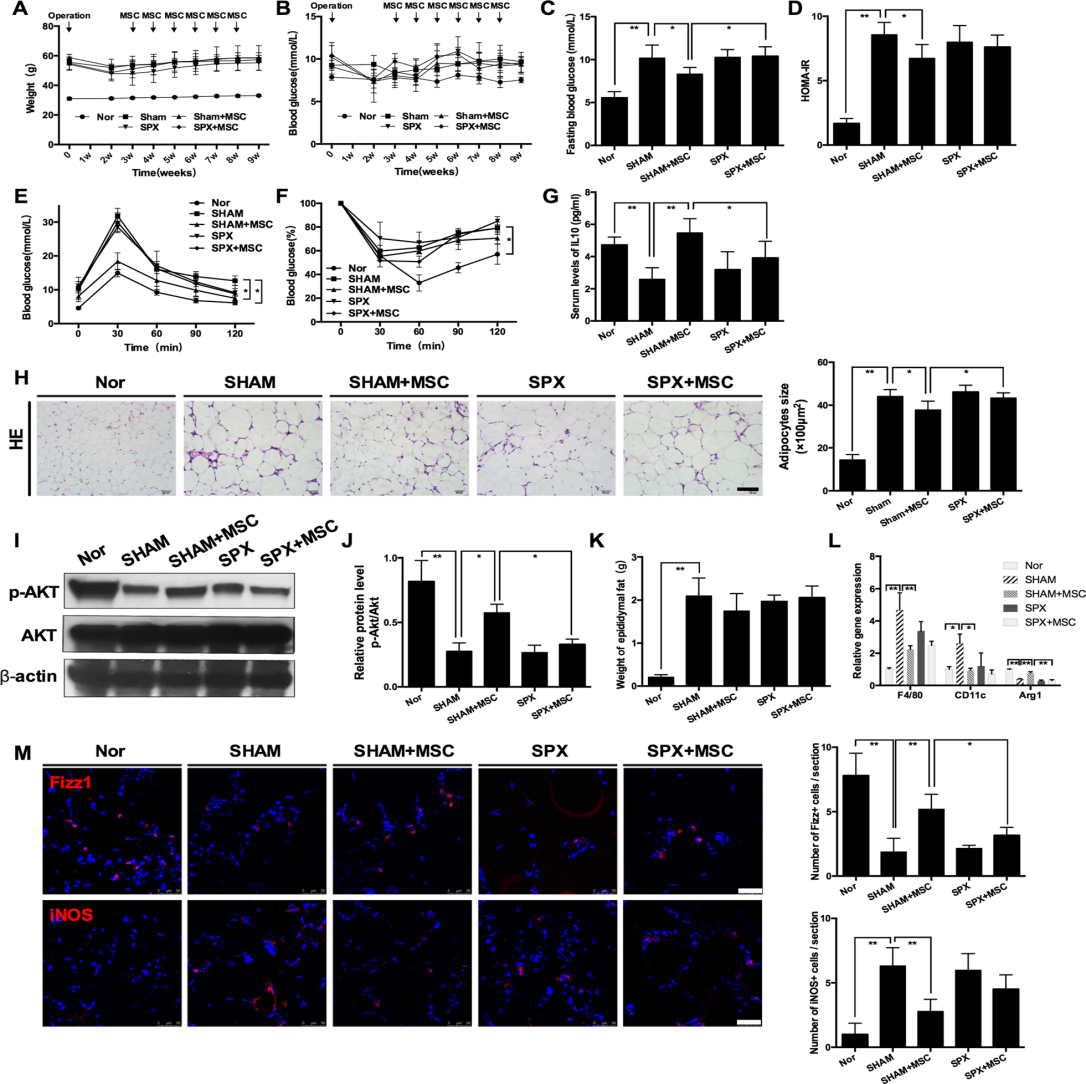
Splenectomy dampened UC-MSC-induced mitigation of insulin resistance in epididymal adipose tissue. HFD-induced obese mice underwent splenectomy or sham operation. After 3 weeks of recovery, mice that underwent splenectomy were infused with UC-MSCs (referred to as the SPX + MSC group) or PBS (referred to as the SPX group) once per week for 6 weeks. Similarly, mice that underwent sham operation were infused with UC-MSCs (referred to as the SHAM + MSC group) or PBS (referred to as the SHAM group) once per week for 6 weeks. Mice fed a normal chow diet were used as controls (referred to as the Nor group). **a** Weight of the mice. **b** Random blood glucose levels of the mice. **c** The fasting glucose levels after the last infusion of UC-MSCs were detected. The HOMA-IR score was calculated (**d**). After the last infusion of UC-MSCs, an IPGTT (**e**) and IPITT (**f**) were performed to evaluate glucose tolerance and insulin tolerance, respectively. **g** Serum levels of IL-10. **h** Representative H&E staining of epididymal adipose tissue; the size of adipocytes was evaluated via digital image analysis. Scale bar = 100 μm. **k** Weight of epididymal adipose tissue. **l** Real-time polymerase chain reaction analysis of macrophage phenotype-related gene expression in epididymal adipose tissue. **i** Immunoblotting analysis of p-AKT and total AKT expression in epididymal adipose tissue; the ratios of p-AKT to total AKT were quantitated (**j**). **m** Representative Fizz1-positive cells and iNOS-positive cells in epididymal adipose tissue analysed by immunofluorescence and quantification of Fizz1-positive or iNOS-positive cells. Scale bar = 50 μm. Values in **m** were determined by assessing cells manually from at least five sections of each slide, at least 3 slides per mouse, and at least 6 mice per group. Values of **a**–**h** are the means ± SDs; *n* = 6 mice per group; *P < 0.05, **P < 0.01

There was no significant difference between the SHAM group and SPX group in body weight (Fig. 6A), systemic metabolic homeostasis (Fig. 6B–F), insulin sensitivity (Fig. 6I, J) or macrophage phenotype of EAT (Fig. 6L, M). Compared with the SHAM group, the SHAM + MSC group displayed an obvious decline in blood glucose levels during the IPGTT (Fig. 6E) and significant decreases in fasting blood glucose levels (Fig. 6C) and HOMA-IR scores (Fig. 6D). Splenectomy partly abolished the effects of UC-MSCs, as the fasting blood glucose levels and blood glucose levels during the IPGTT test of the SPX + MSC group were markedly higher than those of the SHAM + MSC group. Of note, splenectomy blocked the UC-MSC-induced increase in serum IL-10 levels, indicating that spleen-derived IL-10 was an essential source of increasing serum IL-10 levels (Fig. 6G). Further analyses showed that compared with the SHAM + MSC group, the SPX + MSC group displayed significantly larger adipocytes, lower p-AKT expression,lower Arg-1 expression, less Fizz1-positive cells, and an ascending trend in iNOS-positive cells in EAT, implying that splenectomy impaired the effectiveness of UC-MSCs in alleviating insulin resistance and polarizing macrophages in EAT (Fig. 6H–M). In summary, these results suggested that the beneficial effects of UC-MSCs were, at least in part, mediated by the interaction between MSCs and splenocytes in the spleen.

### UC-MSCs increase IL-10 expression in Treg cells in the spleen

**Fig. 7:**
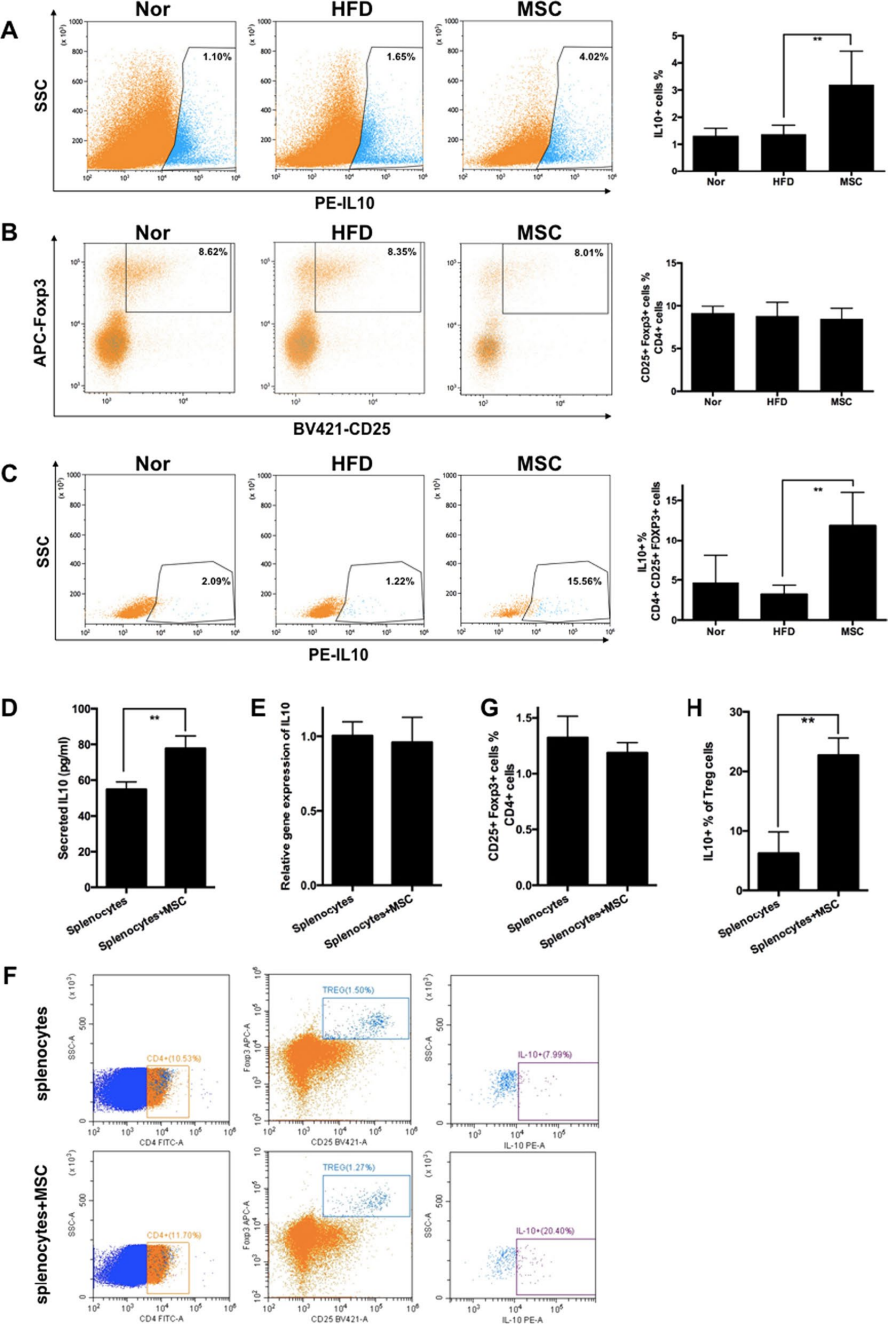
UC-MSCs increase IL-10 expression in Treg cells in the spleen. HFD-induced obese mice were randomly divided into an MSC group (received UC-MSC infusion once per week for 6 weeks) and an HFD group (received corresponding PBS infusions). Mice fed a normal chow diet were used as controls (referred to as the Nor group). One week after the last UC-MSC infusion, the mice were sacrificed, and the spleen was harvested. Then, splenocytes were extracted from the spleen and detected by flow cytometry (**a**, **b**, **c**). **a** The percentage of IL-10^+^ cells among total splenocytes. **b** The percentage of CD25^+^Foxp3^+^ cells among CD4^+^splenocytes. **c** The percentage of IL-10^+^ cells among CD4^+^CD25^+^Foxp3^+^ cells. *n* = 6 mice per group. **d**–**h** Splenocytes collected from HFD-fed obese mice were co-cultured with UC-MSCs via a Transwell system or cultured alone for 24 h, followed by removing the UC-MSCs and stimulating the splenocytes with ConA for 72 h. During the whole period, the splenocytes were cultured with RPMI 1640 medium supplemented with murine recombinant interleukin-2 (20 U/ml). The levels of IL-10 in splenocyte supernatant were detected by ELISA (**d**). The IL-10 gene expression in the splenocytes was detected by real-time polymerase chain reaction analysis (**e**). The percentage of CD25^+^Foxp3^+^ cells among CD4^+^ splenocytes and the percentage of IL-10^+^ cells among CD4^+^CD25^+^Foxp3^+^ cells were detected by flow cytometry (**f**, **g**, **h**). Values are the means ± SDs of three individual experiments, *P < 0.05, **P < 0.01

To further elucidate the source of IL-10 level elevation in the spleen after UC-MSC infusion, we investigated changes in both immune cell profiles and intracellular expression of IL-10 in various types of immune cells after multiple UC-MSC infusions. Flow cytometry showed that the percentage of IL-10-positive cells in the MSC group increased in comparison with that in the HFD group (HFD group vs. MSC group, 1.43% vs. 3.14%, *P* < 0.05) (Fig. 7A). Previous research has shown that the main sources of IL-10 include type 2 helper T cells, Treg cells, B10 cells and macrophages [31, 32]. Excitingly, we found that the percentage of IL-10-positive Treg cells dramatically rose (HFD group vs. MSC group, 3.19% vs. 11.65%, *P* < 0.01) (Fig. 7C) after UC-MSC infusions, but the percentage of Treg cells among CD4 ^+^ T cells was not altered after UC-MSC infusion (Fig. 7B). UC-MSCs did not significantly increase the proportion of IL-10-positive B10 cells (Additional file 1: Fig. 4C, D), CD4 + T cells (Additional file 1: Fig. 5D, E), CD8 + T cells (Additional file 1: Fig. 5F, G), natural killer cells (Additional file 1: Fig. 6J, K), monocytes (Additional file 1: Fig. 6F, G) or neutrophils (Additional file 1: Fig. 6D, E), although the percentage of B10 cells (Additional file 1: Fig. 4A, B) was elevated slightly. The proportion of IL-10-positive M2 macrophages in the spleen increased slightly (Additional file 1: Fig. 3D, E). Given the remarkable increase in the levels of IL-10-positive Treg cells, we believed that the UC-MSC-induced rise in IL-10 expression within the spleen was mainly attributed to the upregulated capacity of IL-10 production by Treg cells.

Next, in an in vitro study, we cocultured splenocytes collected from HFD-fed obese mice with UC-MSCs for 24 h, followed by stimulation of splenocytes with ConA for 72 h. UC-MSCs upregulated the protein level of IL-10 in the supernatant of splenocytes (Fig. 7D), whereas the gene expression level of IL-10 did not change (Fig. 7E), indicating that UC-MSCs probably modified IL-10 expression in splenocytes at the posttranscriptional level. Further analyses by flow cytometry showed that the percentage of IL-10-positive Treg cells increased sharply (control group vs. MSC group, 6.26% vs. 22.71%, *P* < 0.01), coupled with no alteration in the proportion of Treg cells (Fig. 7F–H). In summary, these data indicated that UC-MSC-induced increases in the expression of IL-10 in the spleen were mainly derived from Treg cells.

## Discussion

In this study, we demonstrated that multiple infusions of UC-MSCs were effective in improving metabolic homeostasis and alleviating adipose tissue insulin resistance in HFD-induced obese mice by promoting macrophage polarization towards an anti-inflammatory phenotype. Moreover, UC-MSCs elevated the IL-10 levels in EAT, the spleen and blood, and blockade of IL-10 in vivo remarkably blunted the effects of UC-MSCs. Interestingly, UC-MSCs rarely homed to EAT but largely engrafted to the spleen, and splenectomy abrogated UC-MSC-induced beneficial effect in EAT. Finally, both in vivo and in vitro studies demonstrated that UC-MSCs promoted IL-10 expression in Treg cells in the spleen. It can be concluded that that elevation of spleen-derived IL-10 levels through UC-MSCs interacting with Treg cells at least partially accounted for the protective effect of UC-MSCs on obese mice, indicating the spleen is a pivotal target in UC-MSC therapy for chronic inflammatory diseases.

Inconsistent results have been reported regarding the effect of MSCs on HFD mice. Six doses of intravenously UC-MSCs infusion in our research and six doses of intraperitoneal adipose tissue-derived MSCs (AD-MSCs) infusion in the research by Shang et al. [33] both resulted in mitigation of adipose tissue inflammation and insulin resistance [33]. However, Nyamandi et al. found that two doses of intravenously infused murine bone marrow-derived MSCs (BM-MSCs) failed to elicit significant changes in inflammatory markers and insulin sensitivity in HFD mice [34]. The discrepancy may be attributed to the differences in the sources of MSCs and doses of MSCs. Studies have shown that MSCs from different sources have different multilineage differentiation potentials and immunomodulatory functions [35–38]. Compared with AD-MSCs [36] and BM-MSCs [39], UC-MSCs grew faster in vitro. Moreover, a study by Dabrowski et al. revealed that UC-MSCs displayed higher production of transforming growth factor β and lower production of vascular endothelial growth factor-α than AD-MSCs [38]. In addition, in comparison with AD-MSCs, UC-MSCs showed less expression of human leukocyte antigen DR after stimulation and inhibited the proliferation of peripheral blood mononuclear cells more prominently [36], indicating that UC-MSCs probably had lower immunogenicity and stronger immunomodulatory ability. Previous studies showed that compared with a single MSC infusion, multiple MSC infusions were more effective in alleviating insulin resistance in T2DM mice [40, 41]. The inadequate number of MSC infusions in the study by Nyamandi et al. may account for the unchanged insulin resistance in obese mice. In addition, we found intravenously infused UC-MSCs rarely engrafted to EAT but overwhelmingly and sustainably persisted in the spleen, while research by Qianwen Shang et al. found that intraperitoneally infused AD-MSCs homed to EAT, indicating that different infusion route may result in different underlying mechanism by which MSCs elicited alleviation of insulin resistance.

IL-10 is recognized as a potent anti-inflammatory cytokine and has the ability to induce the differentiation of macrophages into M2 [4, 22, 42]. Both animal experiments [25] and epidemiological data [23, 24] have demonstrated that obesity leads to a systemic reduction in IL-10 levels in obese individuals. In agreement with previous research, we found that serum IL-10 levels and IL-10 expression in EAT decreased in HFD-induced obese mice, coupled with increasing M1 macrophage levels in EAT. Multiple UC-MSC infusions remarkably restored systemic IL-10 levels and increased the number of M2 macrophages in WAT, while in vivo blockade of IL-10 impaired UC-MSC effects. Interestingly, further investigation revealed that the increase in IL-10 levels after UC-MSC infusion was derived from the spleen, as splenectomy abrogated the UC-MSC-induced elevation of IL-10 levels in serum and EAT. Similar to our study, some previous research also reported that MSC infusion elevated systemic IL-10 levels and promoted organ function restoration by upregulating spleen-derived IL-10 expression in an acute spinal injury rat model [17] and T2DM rat model [43]. But in these studies, the explicit link between increasing IL-10 levels and amelioration of organ damage remained unexplored. Notably, our study showed that elevated serum IL-10 levels improves insulin sensitivity in EAT by promoting macrophage polarization. Our previous studies in a long-term T2DM complication rat model showed that multiple MSC infusions increased IL-10 expression and polarized macrophages into the M2 phenotype in the kidney, liver and lung [14]. Hence, we speculate that in inflammatory disease models, the elevation in spleen-derived IL-10 levels induced by MSCs is a critical contributor of a systemic rise in IL-10 levels, which may lead to macrophage polarization in multiple organs. As M2 macrophages have a strong capacity to secrete IL-10, it is possible that the MSC-induced M2 macrophages also contribute to the rise in IL-10 levels. In the future, further research is needed to validate this hypothesis in other inflammatory disease models.

The spleen, as the largest lymphoid organ, plays an important role in the capture and removal of pathogens, erythrocyte turnover, iron recycling, modulation of inflammatory responses and production of chemokines and cytokines [44]. Of note, unlike previous research by Gotoh et al., we noticed that splenectomy alone did not exacerbate obesity or cause a relative reduction in serum IL-10 levels, probably due to differences in experimental procedures, duration of HFD feeding and recovery period. Gotoh et al. performed sham operation or splenectomy on young C57BL/6 J mice and then fed the mice a HFD for 8 weeks, so their study showed that the spleen played an important role in the establishment and early-stage progression of obesity. However, we fed mice a HFD for 20 weeks to establish the obese mouse model and then conducted sham operation or splenectomy on obese mice. Our results demonstrated that the absence of the spleen did not necessarily lead to the exacerbation of established obesity in a short period. Moreover, in our research, the mice underwent splenectomy or sham surgery and then recovered for 3 weeks before UC-MSC infusion to exclude potential external disturbances. Given that bone marrow is also a well-established source of IL-10 [45], during the recovery period, bone marrow may compensate splenic function in cytokine production, leading to unchanged IL-10 levels after splenectomy.

Of note, several recent studies demonstrated that the interaction between MSCs and splenocytes accounted for the preventive or protective efficacy of MSCs on traumatic spinal cord injury [17], acute renal injury [16], and myocardial infarction [46], although few MSCs engrafted to the injured organ in the abovementioned disease models. For example, Hu et al. demonstrated that splenic involvement played a central role in BM-MSC-mediated alleviation of acute kidney injury by increasing the percentages of Treg cells in the spleen. Badner et al. reported that UC-MSC infusions decreased spinal cord haemorrhage and elevated serum levels of IL-10 in a rat model of traumatic spinal cord injury but these UC-MSC-induced effects were markedly impaired after splenectomy. These studies supported the notion that the spleen plays an essential role in MSC treatment of diseases characterized by acute inflammation. Nevertheless, research about splenic involvement in the therapeutic effect of MSCs on diseases featuring chronic inflammation is limited. Our results implied that splenic involvement was crucial in the effect of UC-MSCs on diseases characterized by chronic inflammation, such as obesity. In the future, further attention should be given to the spleen during the exploration of the therapeutic effect of MSCs.

In addition, a previous study demonstrated that in rodent models of T2DM, multiple MSC infusions resulted in macrophage polarization in various organs, including the pancreas, adipose tissue, and kidney, without engraftment into the targeted organ [14, 20]. In other words, it seems that MSC infusion can systemically modulate macrophage phenotypes. Intriguingly, our research revealed that macrophage polarization in EAT was at least partially attributed to UC-MSC-induced splenic production of IL-10. This mechanism may also account for MSC-induced systemic macrophage polarization in other metabolic diseases. Our research contributed to delineating the systemic immunomodulatory effects of MSCs.

A number of studies have demonstrated the immunoregulatory effects of MSCs on Treg cells [47, 48]. For example, González et al. reported that a systemic infusion of MSCs induced and activated CD4^+^CD25^+^Foxp3^+^ Treg cells, coupled with an increase in IL-10 levels in mice suffering from experimental colitis [49]. Madec et al. found that an infusion of MSCs protected NOD mice from developing spontaneous diabetes by promoting the expansion of IL-10-producing Treg cells in the pancreas [50]. Intriguingly, Hu et al. found that MSCs alleviated acute kidney injury by increasing the percentage of Treg cells in the spleen [16]. However, we found that UC-MSCs did not elicit an increase in the percentage of Treg cells in the spleen, probably owing to differences in the disease model. Our research showed that UC-MSCs promoted the capacity of Treg cells to produce IL-10, and in vitro study further confirmed the results, implying that UC-MSCs enhanced the function of Treg cells, although they did not induce the expansion of Treg cells. Under different stimuli, Treg cells undergo specific differentiation leading to unique functional properties. Treg cells can be classified into resting Treg cells and effector Treg cells in peripheral lymphoid organs, and secretion of IL-10 is one of the key features of effector Treg cells [51, 52]. Many transcription factors are involved in the specialized differentiation and production of IL-10 in effector Treg cells. The expression of transcription factor B lymphocyte-induced maturation protein (Blimp1) and the transcription factor interferon regulatory factor 4 (IRF4) is pivotal in the production and secretion of IL-10 in effector Treg cells [53, 54]. UC-MSCs probably elevated the percentage of effector Treg cells and/or induced the expression of Blimp1 and IRF4 in effector Treg cells, thus increasing IL-10 expression in total Treg cells in the spleen. Further investigation is needed to elucidate the underlying mechanism by which UC-MSCs augment the expression of IL-10 in Treg cells.

## Conclusion

We demonstrated that multiple UC-MSC infusions mitigated insulin resistance in EAT of HFD-induced obese mice at least partially by promoting IL-10 production by Treg cells in the spleen. Our observations provide important evidence that MSC-splenocyte interactions play a pivotal role in MSC-induced therapeutic effects. These findings might have important implications for the clinical application of MSCs in the future.

## Supplementary Information

**Additional file 1.** **Table S1.** Primer sequences of target genes (mice). **Fig. S1.** The induction of obese mice. **Fig. S2.** UC-MSC homing in obese mice. **Fig. S3.** The effect of UC-MSC infusions on macrophages in the spleen. **Fig. S4.** The effect of UC-MSC infusions on B10 cells in the spleen. **Fig. S5.** The effect of UC-MSC infusions on CD4^+^ T cells and CD8^+^ T cells in the spleen. **Fig. S6.** The effect of UC-MSC infusions on monocytes, neutrophils and NK cells in the spleen.

## Data Availability

The datasets used and/or analysed during the current study are available.

## Abbreviations

WAT: White adipose tissues
Fizz1: Found in inflammatory zone 1
Arg1: Arginase-1
iNOS: Inducible nitric oxide synthase
TNF-α: Tumour necrosis factor-α
MSCs: Mesenchymal stem cells
UC-MSCs: Human umbilical cord-derived MSCs
T2DM: Type 2 diabetes mellitus
IL-10: Interleukin 10
HFD: High-fat diets
Treg cells: Regulatory T cells
PBS: Phosphate-buffered saline
IPGTTs: Intraperitoneal glucose tolerance tests
IPITTs: Intraperitoneal insulin tolerance tests
ELISA: Enzyme-linked immunosorbent assay
HOMA-IR: Homeostatic model assessment for insulin resistance
CM-Dil: Chloromethyl-benzamidodialkylcarbocyanine
EAT: Epididymal adipose tissue
BSA: Bovine serum albumin
RT-PCR: Real-time polymerase chain reaction
DAPI: 4'-6-Diamidino-2-phenylindole
IL-1β: Interleukin 1β
IL-6: Interleukin 6
MCP-1: Monocyte chemoattractant protein-1
ConA: Concanavalin A
AD-MSCs: Adipose-derived MSCs
BM-MSCs: Bone marrow-derived MSCs
Blimp1: B lymphocyte-induced maturation protein
IRF4: Interferon regulatory factor 4
