# Supplementary material for: Human umbilical cord-derived mesenchymal stem cells alleviate insulin resistance in diet-induced obese mice via an interaction with splenocytes
Source: Stem Cell Res Ther. 2022 Mar 21;13:109. doi: 10.1186/s13287-022-02791-6 (PMC8935757; doi:10.1186/s13287-022-02791-6)
Supplement: Supplementary file 1 — Additional file 1. Table S1. Primer sequences of target genes (mice). Fig. S1. The induction of obese mice. Fig. S2. UC-MSC homing in obese mice. Fig. S3. The effect of UC-MSC infusions on macrophages in the spleen. Fig. S4. The effect of UC-MSC infusions on B10 cells in the spleen. Fig. S5. The effect of UC-MSC infusions on CD4+ T cells and CD8+ T cells in the spleen. Fig. S6. The effect of UC-MSC infusions on monocytes, neutrophils and NK cells in the spleen. [file 13287_2022_2791_MOESM1_ESM.docx]

**Additional file 1**

Table S1 Primer sequences of target genes (mice).

| Genes | Primer sequence(5'-3') |
| --- | --- |
| β-actin | For: AGTGTGACGTTGACATCCGT |
|  | Rev: GCAGCTCAGTAACAGTCCGC |
| IL-10 | For: CAGCTCAGAGGGTTCCCCTA |
|  | Rev: CTGGCCACAGTTTTCAGGGA |
| IL-1β | For: TGGGCCTCAAAGGAAAGAAT |
|  | Rev: CAGGCTTGTGCTCTGCTTGT |
| IL-6 | For: TAGTCCTTCCTACCCCAATTTCC |
|  | Rev: TTGGTCCTTAGCCACTCCTTC |
| TNF-α | For: CCAGACCCTCACACTCAGATC |
|  | Rev: CACTTGGTGGTTTGCTACGAC |
| CD11b | For:GGGTCATTCGCTACGTAATTGG |
|  | Rev:TGTTCACCAGCTGGCTTAGATG |
| Arg1 | For:AGACCACAGTCTGGCAGTTG |
|  | Rev:CCACCCAAATGACACATAGG |
| NOS2 | For:ACCTTGGTGAAGGGACTGAG |
|  | Rev:TCCGTTCTCTTGCAGTTGAC |
| F4/80 | For:CTTTGGCTATGGGCTTCCAGTC |
|  | Rev:GCAAGGAGGACAGAGTTTATCGTG |
| CD11c | For: ACGTCAGTACAAGGAGATGTTGGA |
|  | Rev: ATCCTATTGCAGAATGCTTCTTTACC |


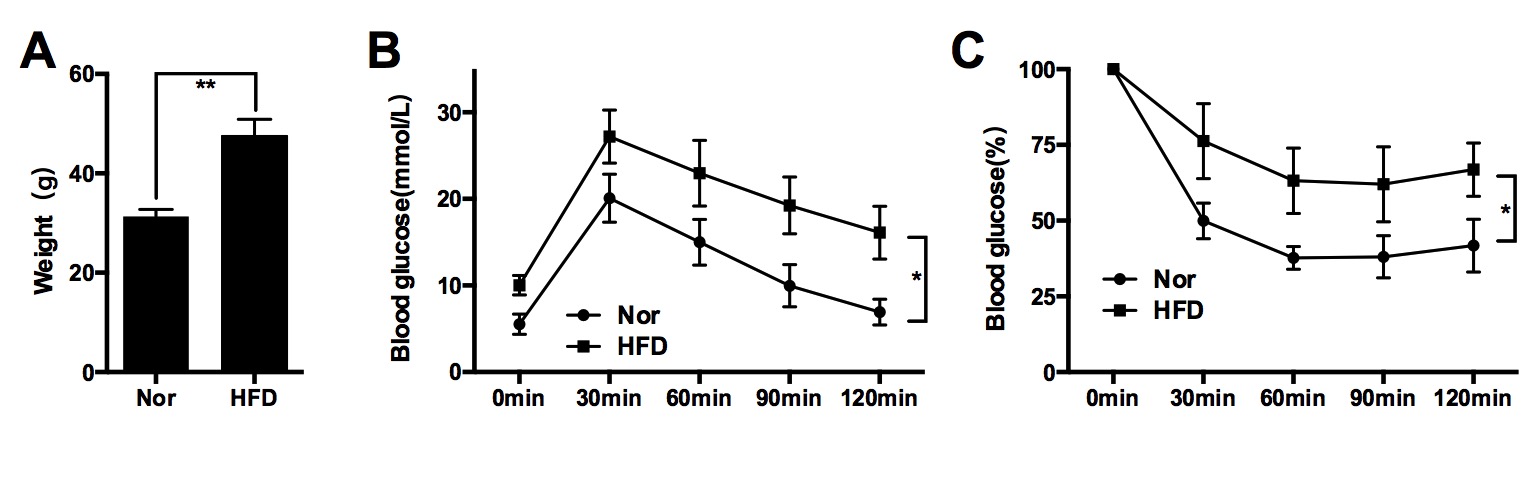


**Fig. S1** The induction of obese mice. Eight-week-old male C57BL/6J mice were fed a HFD for 20 weeks to induce obesity. The weight of mice fed with normal diets (Nor group) or high-fat diets (HFD group) for 20 weeks (**a**). After feeding 20 weeks, glucose tolerance and insulin tolerance were assessed by an IPGTT (**b**) and IPITT (**c**), respectively. n = 6-12 mice per group; *P < 0.05, **P < 0.01.


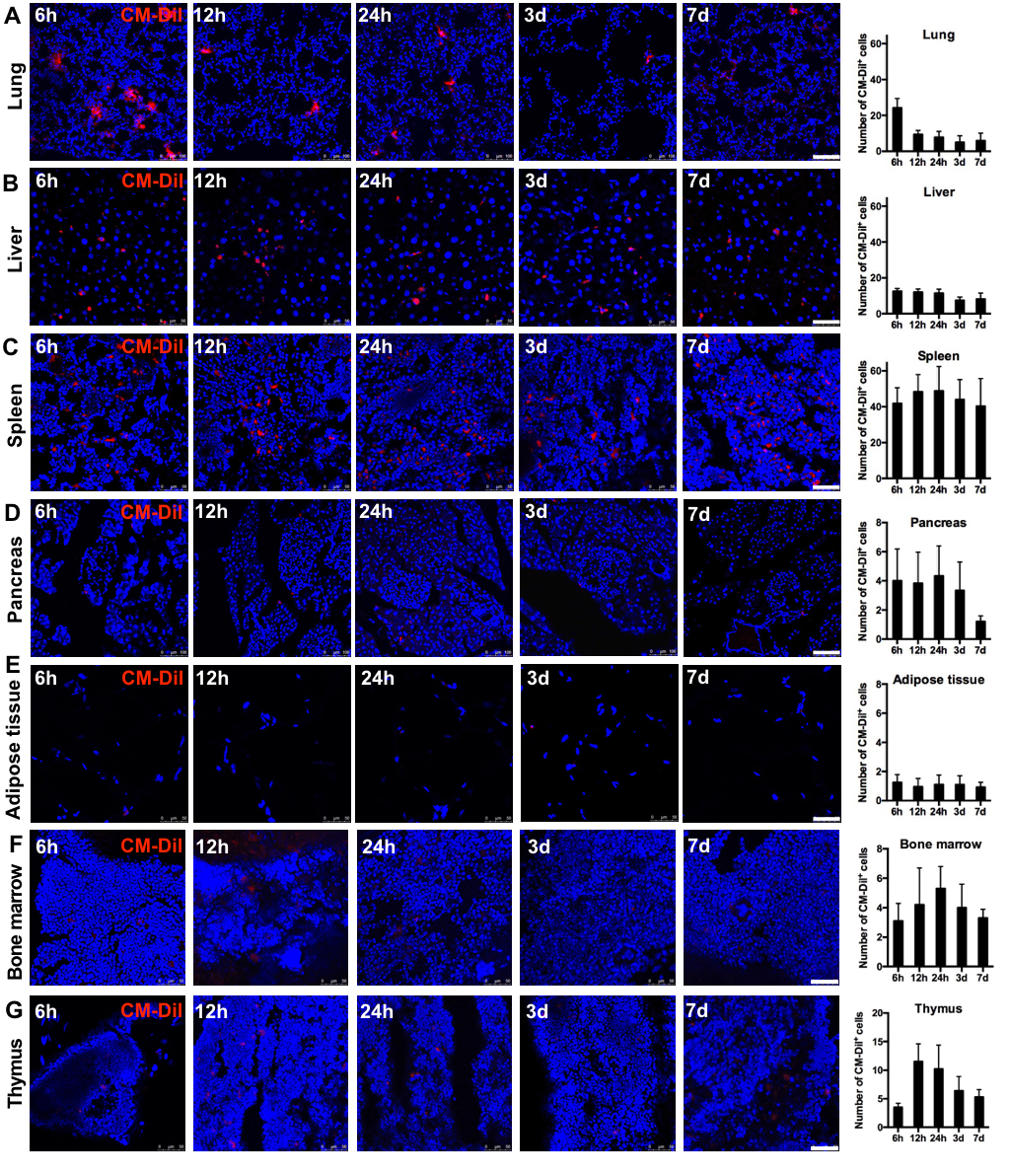


**Fig. S2** UC-MSC homing in obese mice. HFD-induced obese mice were intravenously infused with 1×10^6^ UC-MSCs labeled with CM-Dil before infusion. Then, the mice were sacrificed at 6 h, 12 h, 24 h, 3 d and 7 d after UC-MSC infusions. UC-MSC tracking in the lung (**a**), liver (**b**), spleen (**c**), pancreas (**d**), epididymal adipose tissue (**e**), bone marrow (**f**) and thymus (**g**) was evaluated by confocal laser scanning microscopy. Scale bar, 100 μm (**a, d**), 50 μm (**b, c, e, f, g**). Values are presented as the means ± SDs. n = 6 mice per group; *P < 0.05, **P < 0.01.

**
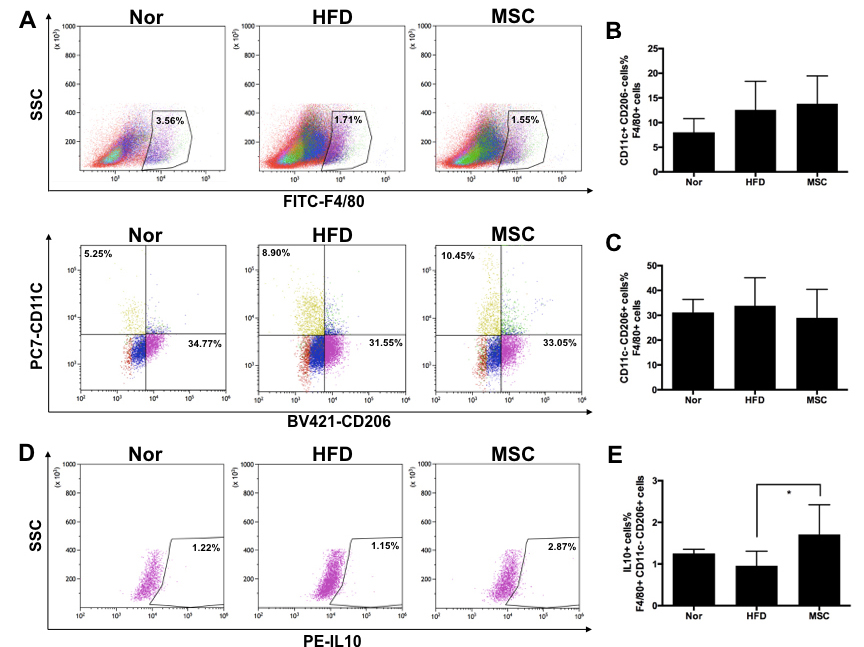
Fig. S3** The effect of UC-MSC infusions on macrophages in the spleen. One week after the last UC-MSC infusion, the mice were sacrificed, and the spleen was harvested. Then, splenocytes were extracted from the spleen and detected by flow cytometry. **a** Freshly obtained splenocytes were incubated with F4/80-FITC, CD11c-PC7, CD206-BV421 and IL-10-PE antibodies. F4/80^+^ cells were selected for CD11c^+^ and CD206^+^ screening. The proportions of CD11c^+^ CD206^-^ and CD11c^-^ CD206^+^ cells are shown in **b** and **c**, respectively. **d, e** The proportion of IL-10^+^ cells among F4/80^+^ CD11c^-^ CD206^+^ cells was detected by flow cytometry.

**
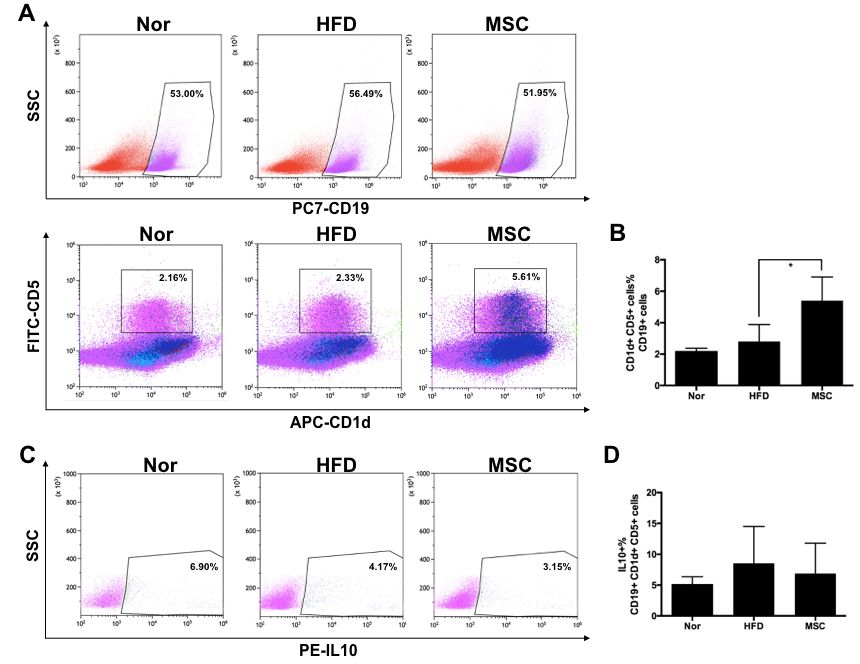
Fig. S4** The effect of UC-MSC infusions on B10 cells in the spleen. One week after the last UC-MSC infusion, the mice were sacrificed and the spleen was harvested. Then, splenocytes were extracted from the spleen and detected by flow cytometry. **a** Freshly obtained splenocytes were incubated with CD19-PC7, CD1d-APC, CD5-FITC and IL-10-PE antibodies. CD19^+^ cells were selected for CD1d ^+^ and CD5^+^ screening. The proportion of CD1d ^+^ CD5^+^cells among CD19^+^ cells is shown in **b. c, d** The proportion of IL-10^+^ cells among CD19^+^ CD1d ^+^ CD5^+^ cells was detected by flow cytometry. Values are presented as the means ± SDs. n = 6 mice per group; *P < 0.05, **P < 0.01.

**
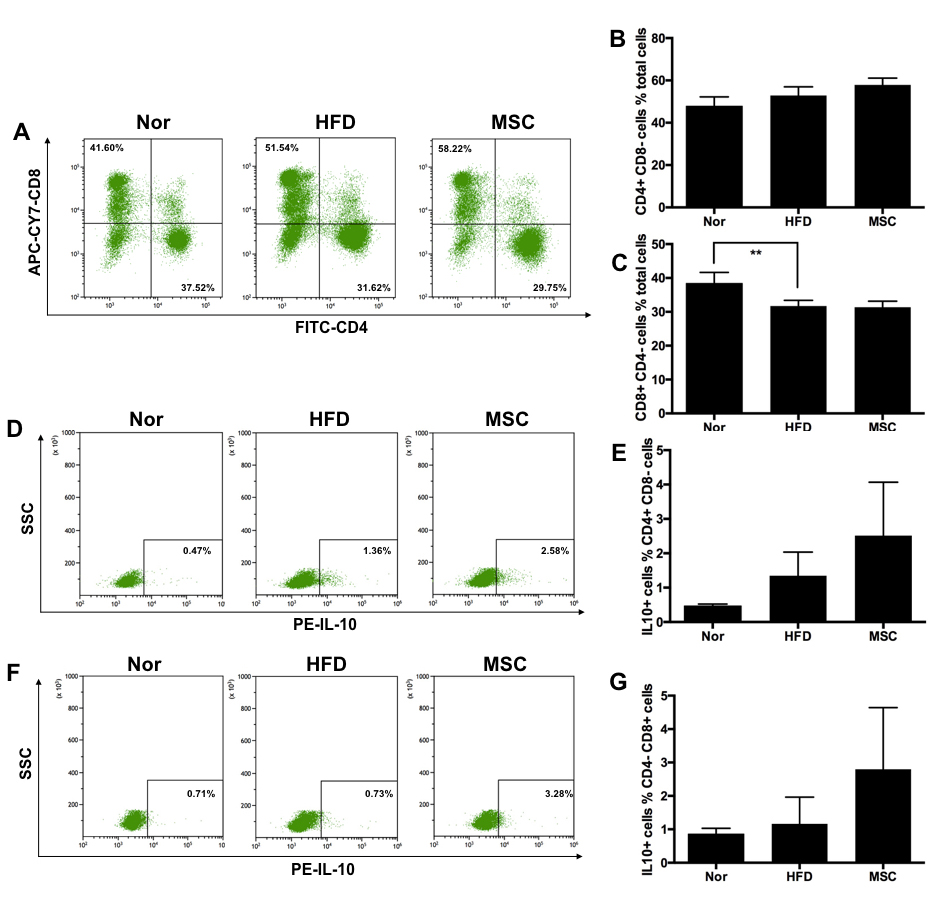
Fig. S5** The effect of UC-MSC infusions on CD4^+^ T cells and CD8^+^ T cells in the spleen. One week after the last UC-MSC infusion, the mice were sacrificed and the spleen was harvested. Then, splenocytes were extracted from the spleen and detected by flow cytometry. Freshly obtained splenocytes were incubated with CD3-BV421, CD4-FITC, CD8-APC-CY7 and IL-10-PE antibodies. **a** The expressions of CD4 and CD8 were detected by flow cytometry. The proportions of CD4^+^CD8^-^ and CD4^-^CD8^+^ cells are shown in **b** and **c**, respectively. **d, e** The proportion of IL-10^+^ cells among CD4^+^ CD8^-^ cells was detected by flow cytometry. **f, g** The proportion of IL-10^+^ cells among CD4^-^ CD8^+^ cells was detected by flow cytometry. Values are presented as the means ± SDs. n = 6 mice per group; *P < 0.05, **P < 0.01.


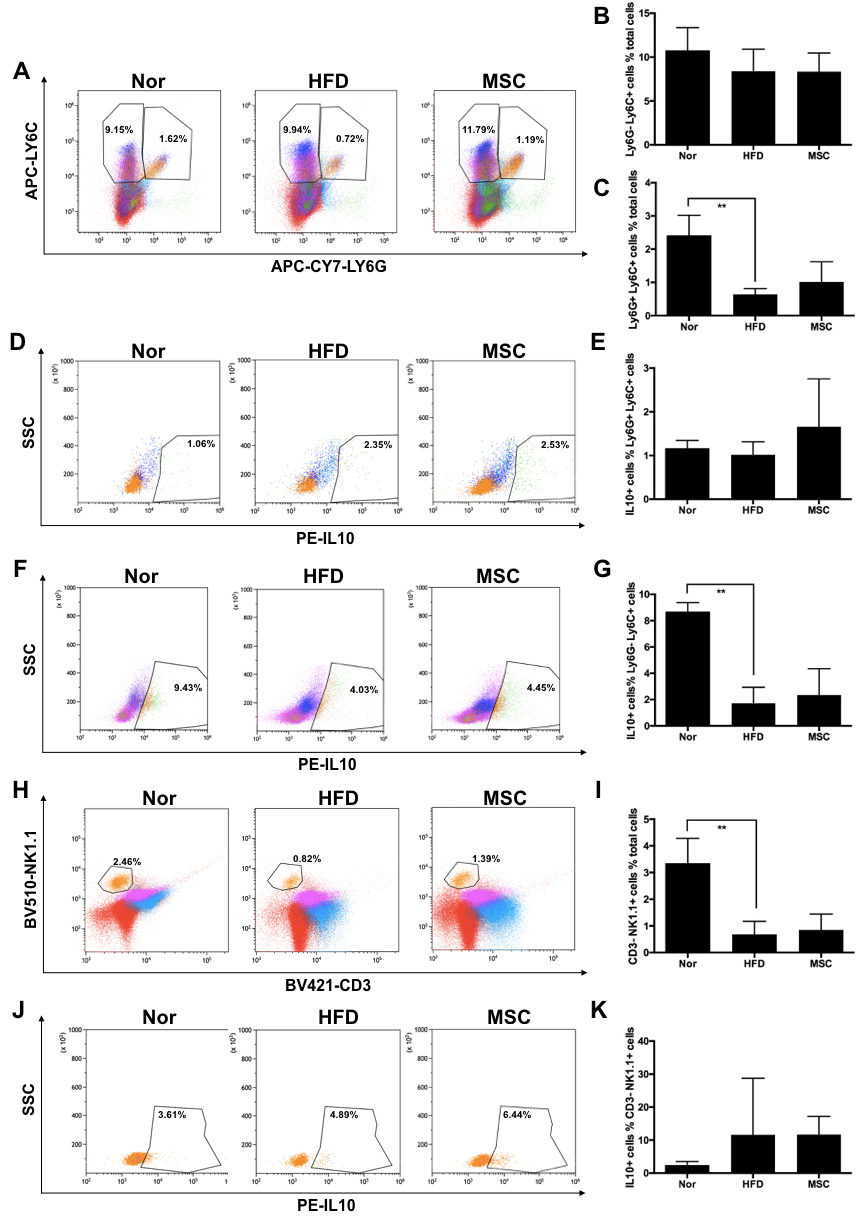
**Fig. S6** The effect of UC-MSC infusions on monocytes, neutrophils and NK cells in the spleen. One week after the last UC-MSC infusion, the mice were sacrificed and the spleen was harvested. Then, splenocytes were extracted from the spleen and detected by flow cytometry. Freshly obtained splenocytes were incubated with LY6G-APC-CY7, LY6C-APC and IL-10-PE antibodies (**a-g**). **a** The expressions of LY6G and LY6C were detected by flow cytometry. The proportion of LY6G^-^LY6C^+^ cells is shown in **b**. The proportion of LY6G^+^LY6C^+^ cells is shown in **c**. **d, e** The proportion of IL-10^+^ cells among LY6G^+^LY6C^+^ cells was detected by flow cytometry. **f, g** The proportion of IL-10^+^ cells among LY6G^-^LY6C^+^ cells was detected by flow cytometry. Freshly obtained splenocytes were incubated with CD3-BV421, NK1.1-BV510 and IL-10-PE antibodies (**h-k**). The proportion of CD3^-^ and NK1.1^+^ cells is shown in **h** and **i**. The proportion of IL-10^+^ cells among CD3^-^ NK1.1^+^ cells is shown in **j** and **k.** Values are presented as the means ± SDs. n = 6 mice per group; *P < 0.05, **P < 0.01.
